# Evolutionary analysis of resident groups’ intention to participate in green retrofit PPP projects of traditional apartment complexes

**DOI:** 10.1038/s41598-023-27626-z

**Published:** 2023-01-19

**Authors:** Yaohong Yang, Ruicong Sun, Yonghao Wang, Mengjuan Zhu, Peishu Yang

**Affiliations:** 1 School of Water Conservancy, North China University of Water Resources and Electric Power, Zhengzhou, 450046 China; 2 Double Carbon Industry Research Institute Co. Ltd., China State Construction Engineering Corporation, Shanghai, 200025 China; 3 College of Arts, Humanities and Social Science, University of Edinburgh, Edinburgh, EH8 9YL UK

**Keywords:** Environmental social sciences, Sustainability

## Abstract

Green retrofit PPP projects of traditional apartment complexes play an important role in promoting the green and low-carbon transformation of the construction industry and achieving China's "double carbon" goals. The integrated retrofit of apartment complexes presupposes that the resident groups agree to the retrofit. Therefore, it is necessary to study the evolutionary mechanism of residents' intention to green retrofit and the transformation process of their behavior, and to explore how to enhance residents' intention to participate. First, the dissemination model of residents' intention to green retrofit is constructed. Then, the strategic interaction among government, social capitals and residents under the PPP model is introduced into the dissemination model to define the state transformation probability of resident groups. Finally, the evolution laws of residents' intention to green retrofit are analyzed. The results show that: (1) the behavior of government regulation and social capitals' effort to retrofit can motivate the number of the resident agreeing to green retrofit to meet the proportional limit, (2) the faster the government chooses the strategy of regulation and the social capitals choose the strategy of effort to retrofit, the faster the number of residents agreeing to green retrofit reaches a steady state, (3) when the level of government publicity and education is too low, the cost of government regulation or the subsidy given to residents is too high, the green retrofit of traditional apartment complexes cannot be achieved. The research conclusions can provide a reference for the government to formulate green retrofit policies.

## Introduction

Global warming caused by human greenhouse gas emissions has brought a huge impact on the economic and social development of many countries. In China, traditional residential buildings are a major component of building stock and energy consumption^1^. From the perspective of China's building area stock, the national building area is 66 billion m^2^ in 2020, of which urban residential building area is 29.2 billion m^2^ and rural residential building area is 22.7 billion m^2^, which together account for more than 78%; from the perspective of China's building operation energy consumption, the national building operation energy consumption is 1.06 billion tce in 2020, of which urban residential building operation energy consumption is 267 million tce and rural residential building operation energy consumption is 229 million tce, which together account for more than 46%^2^. At the same time, China's construction industry will not reach carbon peaking until 2037 under current development conditions^3^. This shows that the current situation of traditional buildings, which consume a lot of energy, must be changed in order to promote China's "double carbon" strategy. To solve the high emission of traditional residential buildings, green retrofits (GRs) are an important way^4^. Studies have shown that, compared with demolition and reconstruction, GRs of traditional residential buildings can not only reduce economic costs, but also better meet the sustainable development requirements of the construction industry^5,6^. Therefore, in order to deal with climate risks and achieve China's "double carbon" goals, it is urgent to further promote the market-oriented development of GR of traditional residential buildings under the intensifying global climate crisis.

GRs show unique advantages in low-carbon, environmental protection, improving energy efficiency, and creating a healthy living environment, etc. However, the government's financial investment is insufficient and technical skills are lacking in the face of such a large stock of traditional residential buildings. To solve this problem, public–private partnership (PPP) has become an important model for implementing GRs of traditional residential buildings^7^. Under the PPP model, the government can effectively relieve financial pressure relying on social capitals to provide high-quality professional services. In this way, it brings residents a more direct and comprehensive sense of well-being and satisfaction while safeguarding the quality of their healthy lives^8^. Traditional residential buildings include traditional apartment complexes (TACs) and traditional single-family residential buildings. The GR of traditional single-family residential buildings can only consider the individual intentions of residents. In contrast, TACs refer to a closed group of buildings containing apartments, where the ownership is belonged to individual residents. Therefore, TACs are usually integrated retrofit. Therefore, it is necessary for residents to vote together, and only when the number of residents who agree to the retrofit exceeds a certain proportion can the retrofit be carried out. Apartment complexes are typical features of urban residential buildings in China due to population and development history, but rich practical and theoretical studies in many countries for retrofit of traditional buildings provide valuable references for GR of TACs, such as the Energy Policy Act in the US, the Building Retrofit Energy Efficiency Financing Scheme in Singapore, the Green Deal in the UK^6^, and GR technologies^9^, performance assessment^10^, etc. This study takes the resident groups in TACs as the research object, and the evolution mechanism and behavior change process of residents' intention to green retrofit (IGR) are emphatically analyzed. Existing studies have pointed out that residents are not a homogeneous group^11,12^, and their intention to participate is affected by internal factors such as their own cognition and judgment, as well as external factors such as the strategic choices of the government and social capitals^13^. However, there is no in-depth analysis of the comprehensive mechanism of different factors on the residents' IGR, and the uniqueness of the GR of TACs and the individual differences within the resident groups are also ignored. In fact, the evolution process of residents' IGR is the result of the interaction between the behavior of individuals within the group and multiple subjects outside the group^14^. (1) From the perspective of multiple players. The main stakeholders of GR PPP projects of TACs are the government, social capitals and residents. The government takes improving the environment and reducing financial pressure as the starting point, and takes guiding the balance of supply and demand and smooth operation of GR market as the main duty. The government changes the strategy of regulation according to the balance of regulatory costs and benefits. Social capitals, as the main supply body of GR market of TACs, have the main goal of investing and retrofitting TACs to obtain maximum benefits. But it will reduce the motivation of social capitals' effort to retrofit in the situation of lack of demand and high retrofit cost. Residents, as consumers, will directly stimulate the GR market with their retrofit demand. Residents will not participate in reform when their retrofit benefits are not fully guaranteed. It can be seen that the government, social capitals and residents are tri-partners with different purposes in GRs. The synergistic development of these three parties promotes the stability of the GR system. Therefore, the dynamic game process among the parties has an impact on the evolution process of the residents' IGR. (2) From the perspective of resident groups. Although energy saving and emission reduction are social responsibilities that every resident needs to undertake, the differences in residents' environmental cognition and value orientation towards environmental protection make it difficult to coordinate and unify residents' IGR in TACs. In addition, the communication and interaction among residents are closer, and the expression of individual intention is easily disseminated widely within the resident group, which has an impact on the evolution process of the residents' IGR. In summary, not only do the individuals within the resident group influence each other, but the strategic choices made by the government and social capitals based on their own interests also influence the residents' IGR. Therefore, this study aims to explore how to improve residents' intention to participate and reach the minimum threshold of the proportion of residents who agree to GR by analyzing the evolutionary mechanism and behavior change process of residents' IGR.

## Literature review

### Factors influencing residents' IGR

In the GR of traditional residential buildings, the government, social capitals and residents are the most important stakeholders. As consumers, residents are the main factors to realize the GR of TACs. Therefore, the GR of TACs is closely related to residents' intention^15^. The change of residents' behavior is a multi-stage dynamic process in which knowledge and awareness play an important role. Guilt can drive changes in residents' energy-related behavior if residents have sufficient knowledge and awareness of environmental and climate issues^16,17^. The role of government policy is crucial to the impact of individuals and has been proved in existing studies^18,19^. Legal policies strongly guide residents' internal factors and indirectly affect residents' IGR^13^; information policies can help residents acquire the latest relevant policies and knowledge of GRs^20^; fiscal incentive policies and the deterrent effect of supervision are indispensable in the implementation of GRs^21,22^, because of the increased economic burden, residents are often discouraged from GR^23^. In addition, residents' IGR is also affected by many factors, such as technology^24^, interest demands^25^, age^26^, geographical location, residential area, residential structure and purpose^27^, etc. The above literature analyzes the factors that affect residents' IGR from the perspective of theoretical or empirical research, but ignores the comprehensive mechanism of different influencing factors on the dissemination of IGR within residents.

### The evolutionary laws of strategies among stakeholders

The GR of traditional buildings is the result of strategic interaction among stakeholders. Scholars have analyzed the decision-making problems of the government, investment retrofitting enterprises, owners and other stakeholders based on evolutionary games. Based on the game analysis between the government and developers in the decision-making process of the GR of existing large-scale public buildings, it is found that the government makes the GR stagnate by coercive means^28^. The reasons for owners and renters refusing to retrofit were revealed according to the game analysis of these two parties in different scenarios^29^. By analyzing the relationship between the government, Energy Service Companies (ESCO) and owners, it was found that the behavioral orientations of the three parties interacted with each other^30^.Government regulation, cost and benefit were the key factors influencing the decision of the players^31^. The strength of government incentives significantly affected the proportion of ESCO and owners participating in the retrofit. The government increased incentives within a reasonable range, which not only improved the returns of ESCO and owners^32,33^, but also solved the financing dilemma of ESCO in GRs^34^. For the lack of ESCO implementation capacity, effective measures to achieve regulatory effectiveness management goals, such as optimization of the government regulatory system, were proposed based on the analysis of key factors affecting the effectiveness of government regulation^35^.

The traditional government-led model of GRs has hindered the sustainable development process of traditional buildings in China. At present, the PPP model is widely applied in highways^36^, water conservancy projects^37^, new energy power infrastructure^8^, charging infrastructure^38,39^, smart cities^40^, public rental housing^41^, etc. It also provides ideas for accelerating the GR of TACs under the PPP model. Nonetheless, the game of interests among cooperative players is still the main problem facing the retrofit. Although changes in a single variable can affect the behavioral choices of stakeholders, the fact that stakeholders reach a stable cooperative relationship depends on the interaction of multiple variables^42^. Cao et al. analyzed the conditions to reach collaboration between the government and social capitals by constructing an evolutionary game model^43^. Social capital changed its evolutionary characteristics to maximize response to GR PPP projects of traditional buildings when the government adopted incentives^44^. Cost reduction and efficiency improvement would promote GRs of PPP reconstruction of buildings was found in the evolution of the strategies of government groups to encourage GRs and social capitals to implement GRs. However, this incentive was easily constrained by objective conditions, compared to the combination of positive and negative incentives policy incentives would be the best measure^45^. All the above studies assume that residents are non-differentiated individuals. In fact, residents' behavioral characteristics are often diverse, and the traditional evolutionary game theory cannot accurately describe the differences in residents' IGR.

### Carbon trading for the construction industry

Carbon trading, as one of the most efficient market mechanisms, has been widely implemented to encourage carbon reduction in many countries^46^. Studies on the behavioral strategies of players in the carbon trading market have focused more on the emission reduction effects and strategic choices of enterprises^47^ and manufacturers^48^ at the production side, retailers^49^ at the sales side, and households^50^ at the consumption side under the changes of carbon emission-related indicators such as emission allowances, carbon prices, and the government's regulatory costs^51^. However, the fact that the construction industry accounts for the largest share of carbon emissions suggests that it should also be included in the carbon trading market to reduce carbon emissions^52^. As early as 2005, the European Union established the first transnational emissions trading scheme, the European Union Carbon Emissions Trading Scheme and it covers the construction industry. In 2009, the US established the Regional Greenhouse Gas Initiative, which is a mandatory carbon trading scheme and stipulates carbon reduction in the construction industry^53^. In 2010, for the purpose of construction industry carbon reduction, the Japanese government established the Tokyo Emissions Trading Scheme^54^. Other regions or countries, such as New York City and Germany, have also included the construction industry in carbon trading to play a role in carbon reduction^55^. Compared with developed countries, China is late in implementing carbon trading. Currently, only the power industry has been included in the national carbon market and the construction industry is not fully covered by the pilot regions in China^56^. China's carbon trading mechanism has not yet been completed, but the global carbon market has accumulated a lot of experience so far, which can provide a reference for China to design its carbon trading mechanism and promote the development of carbon trading in China. Existing scholars have studied the mechanism design^57^, allowance scheme design^58^ and baseline design^59^ of carbon trading for public buildings^60^, residential buildings^61^ and green buildings^62^. For example, Lou et al. found that by including the US construction industry in carbon trading, owners could obtain economic benefits to reduce carbon emissions^46^. Song et al. found that penalties, subsidies and public supervision under regulation policy of carbon trading are more conducive to emission reduction by building owners than the traditional means of administrative intervention by the government^55^. Su analyzed the feasibility of integrating carbon trading into public building contract energy management projects and found that carbon trading into public building contract energy management projects can not only expand low-cost financing channels for retrofits and increase energy efficiency benefits, but also reduce external risks of retrofit projects. It motivated ESCO and public building owners to participate, and the scale effect of public building energy retrofit was formed^63^. Obviously, it is significant to integrate carbon trading into the GR PPP projects of TACs. The participation of residents is an important part of the carbon trading market, and their accurate knowledge of carbon trading is closely related to their enthusiasm for participation^64^. However, these studies have not integrated carbon trading into the GR PPP projects of TACs to analyze the impact of carbon trading on the decision-making of game players.

### Application of dissemination models

The dissemination models are used to simulate the dissemination of biological viruses such as infectious diseases, mainly including $SI$^65^, $SIS$^66^, $SIR$^67^, $SIRS$^68^, $SEIR$^69^ and other models. Later, scholars have conducted studies with the help of dissemination models based on different application contexts and management practices. For example, there are studies on the dissemination of network public opinion on major projects^70^, the dissemination of COVID-19 along traffic routes^71^, the influence of individual risk awareness on the dissemination of diseases^72^, the dissemination of passenger panic in subway emergencies in a self-organized circumstance^73^, the dissemination of self-injurious behavior among adolescents^74^, the dissemination of tacit knowledge within enterprises^75^, and the influence of the evolution of different views of users on information dissemination in online social networks^76^. The individual residents in TACs do not exist in isolation, but are deeply integrated with other individuals and the social environment. Therefore, the behavioral evolution process of the resident group is inextricably linked with the information dissemination and behavioral interaction among individual residents. Therefore, the dissemination model can be applied to study the evolutionary law of the resident group's IGR of TACs.

### Summary

**Figure 1:**
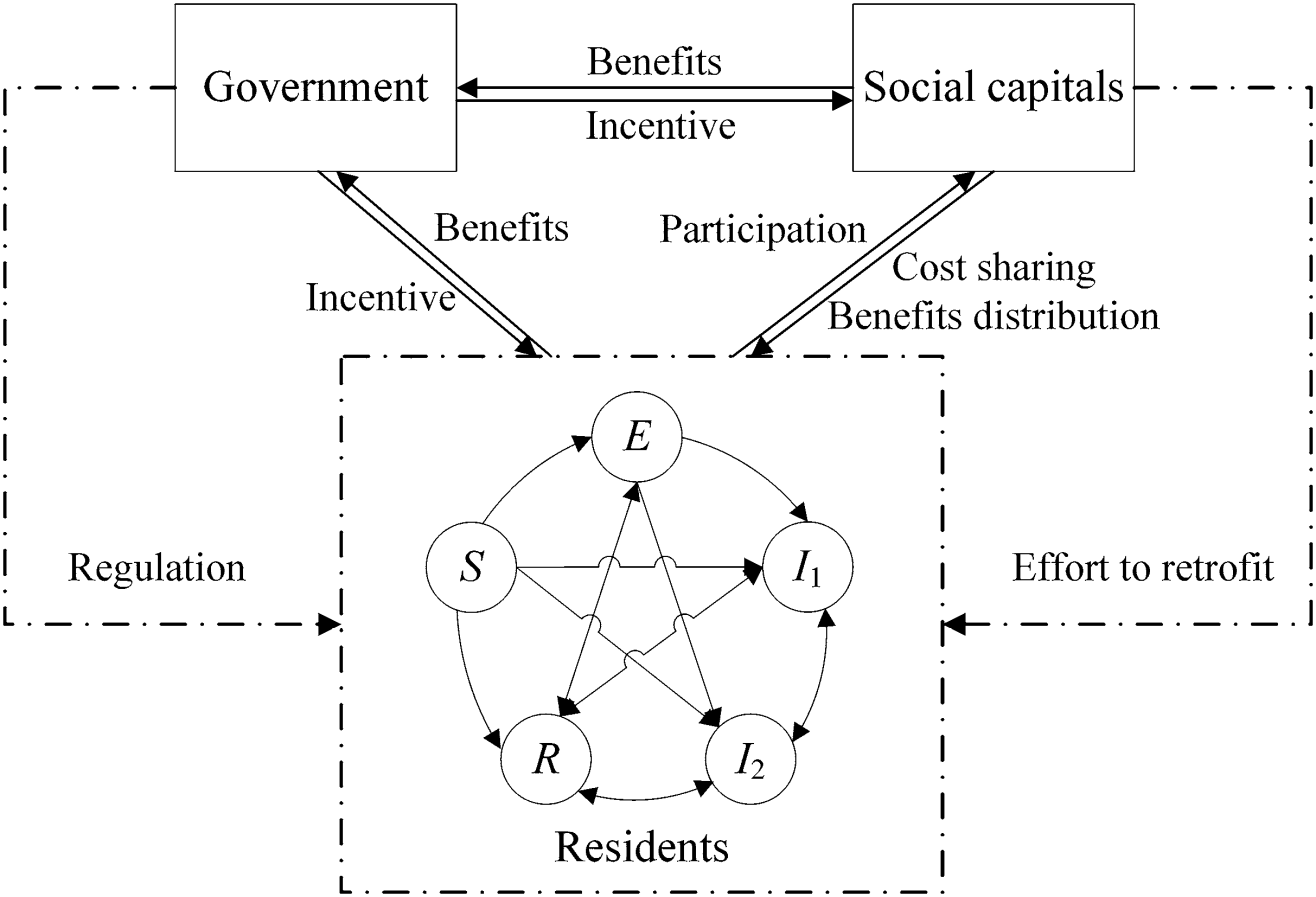
Analytic framework.

First, the existing literature has assumed that residents are homogeneous when studying the game process among stakeholders in GRs of traditional buildings. This indicates that residents do not differ in terms of environmental awareness, retrofitting demands, etc., which is obviously inconsistent with the real situation. Lack of focus on GRs of TACs, as ignoring the differences in residents' IGR affects the implementation of GRs of TACs. Second, the existing literature has analyzed the influencing factors of residents' IGR in depth. However, the synergistic mechanism of different influencing factors on the dynamic dissemination of residents' IGR has been overlooked. Third, existing studies represent the influence of the behavior of the government and social capitals on residents' IGR as exogenous parameters that do not change over time. In fact, there is a complicated game relationship between the government, social capitals and residents, and the whole dynamic game process has different impacts on the evolution process of residents' intention. Therefore, the influences of the government and social capitals on residents' IGR are both time-varying variables. The resident groups' IGR is changing based on the dynamic evolution process of the tripartite. Therefore, the dissemination model of residents' IGR is constructed based on the comprehensive consideration of the influencing factors, which is different from the improved dissemination model in the existing studies. Then, the improved dissemination model is coupled with the evolutionary game model of the government, social capitals and residents in GRs of TACs under the PPP model, in order to define the state transformation probability of the resident group using the evolutionary game model. On this basis, this study explores the influence of the dynamic evolutionary process of the government's and social capitals' strategies on the evolutionary trend of the resident group's IGR, and also analyzes the trajectory of the change in the proportion of residents of each state in TACs under different scenarios. The results of this study provide theoretical references for the government to effectively guide the resident groups to participate in GRs and deeply promote the development of GR PPP projects of TACs. An analytic framework is described in Fig. 1.

## Method

### Construction of the dissemination model

In this paper, the improved SEIR dissemination model is developed to study the dissemination law of residents' IGR of TACs based on the traditional dissemination model.

#### Basic assumptions

##### Assumption 1

Residents in TACs are divided into the following five states: unknown residents $\left( S \right)$ who have not been exposed to GRs; neutral residents $\left( E \right)$ who have been informed of GRs through the dissemination behavior of surrounding residents, but have not disseminated information of GRs to others because they cannot immediately determine a strategy due to their own interests; participating residents $\left( {I_{1} } \right)$ who support GRs and choose to disseminate the positive information of GRs; resistant residents $\left( {I_{2} } \right)$ who oppose GRs and choose to disseminate the negative information of GRs; and immune residents $\left( R \right)$ who have been exposed to GR but do not disseminate or are not affected by the information of GRs because of the decay of interest in GRs. At time $t$, the proportions of unknown, neutral, participating, resistant, and immune residents in the population of residents who live in TACs are represented by $S\left( t \right)$, $E\left( t \right)$, $I_{1} \left( t \right)$, $I_{2} \left( t \right)$, and $R\left( t \right)$, respectively, and satisfy $S\left( t \right) + E\left( t \right) + I_{1} \left( t \right) + I_{2} \left( t \right) + R\left( t \right) = 1$. The meanings of the relevant parameters in the dissemination model are shown in Table 1.

**Table 1:** Relevant parameters in the dissemination model.

Parameters	Meanings	Parameters	Meanings
$\alpha$	The probability of an unknown resident contacting a participating resident	$\gamma$	The probability of an unknown resident contacting a resistant resident
$\beta_{1}$	The success rate of an unknown resident being transformed into a participating resident	$\varepsilon_{1}$	The success rate of an unknown resident being transformed into a resistant resident
$\beta_{2}$	The success rate of an unknown resident being transformed into a neutral resident is influenced by participating residents	$\varepsilon_{2}$	The success rate of an unknown resident being transformed into a neutral resident is influenced by resistant residents
$m_{1}$	The degree of trust of participating residents in resistant residents	$b_{1}$	The level of environmental awareness of the participating residents
$m_{2}$	The degree of trust of resistant residents in participating residents	$b_{2}$	The level of environmental awareness of the resistant residents
$m_{3}$	The degree of trust of immune residents in participating residents	$b_{3}$	The level of environmental awareness of the neutral residents
$m_{4}$	The degree of trust of immune residents in resistant residents	$b_{4}$	The level of environmental awareness of the immune residents
$q$	The level of government publicity and education	$h$	Social climate level
$x$	The probability that the government chooses regulation	$y$	The probability that social capitals choose effort to retrofit

##### Assumption 2

Referring to the study of Zhang et al.^77^, the transformation of unknown residents is influenced by the transformation success rate and the probability of contact with participating and resistant residents. Therefore, an unknown resident may have a strong intention to participate and be transformed into a participating resident with a probability of $\alpha \beta_{1}$ after being exposed to the information of GRs disseminated by participating residents, or generate resistance and be transformed into a resistant resident with a probability of $\gamma \varepsilon_{1}$ after being exposed to the negative information of GRs disseminated by resistant resident, or be transformed into a neutral resident with a probability of $\alpha \beta_{2}$ or $\gamma \varepsilon_{2}$ after being exposed to participating residents or resistant residents due to the consideration of economic benefits, or be transformed into an immune resident with a probability of $\alpha \left( {1 - \beta_{1} - \beta_{2} } \right)$ or $\gamma \left( {1 - \varepsilon_{1} - \varepsilon_{2} } \right)$ directly after being exposed to participating residents or resistant residents without feeling about GR. A neutral resident is transformed into a participating resident with the probability of $z_{1}$, into a resistant resident with the probability of $z_{2}$ or into an immune resident with the probability of $1 - z_{1} - z_{2}$ under the influence of the surrounding environment. A resistant resident and an immune resident are affected by the behavior of the government regulation and social capitals' effort to retrofit with probabilities of $m_{2} xy$ and $m_{3} xy$ into a participating resident, respectively, and a participating resident and an immune resident are also affected by the dissemination of negative information and the behavior of no government regulation and no social capitals' effort to retrofit with probabilities of $m_{1} \left( {1 - x} \right)\left( {1 - y} \right)$ and $m_{4} \left( {1 - x} \right)\left( {1 - y} \right)$ into a resistant resident, respectively. A participating resident and a resistant resident may also lose interest in GRs, being transformed into an immune resident with probability $\left( {1 - b_{1} } \right)\left( {1 - q} \right)$ and $b_{2} q$, respectively. Due to the continuous dissemination of information on GR, an immune resident is transformed into a neutral resident with a probability of $b_{4} h$ given its own environmental awareness and strong social atmosphere.

#### Model construction

**Figure 2:**
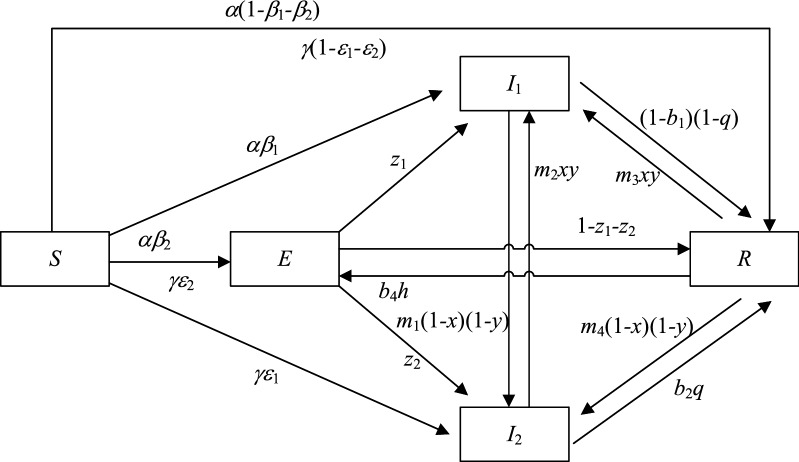
The SEIR dissemination model of residents' IGR.

The transformation process between the residents of each state is represented in Fig. 2.

Accordingly, the corresponding SEIR dissemination dynamic differential equations of residents' IGR of TACs are established as follows:

1 $$ \left\{ \begin{gathered} \frac{dS\left( t \right)}{{d\left( t \right)}} = - \alpha \beta_{1} S\left( t \right)I_{1} \left( t \right) - \gamma \varepsilon_{1} S\left( t \right)I_{2} \left( t \right) - \alpha \beta_{2} S\left( t \right) - \gamma \varepsilon_{2} S\left( t \right) - \alpha \left( {1 - \beta_{1} - \beta_{2} } \right)S\left( t \right) - \gamma \left( {1 - \varepsilon_{1} - \varepsilon_{2} } \right)S\left( t \right) \\ \frac{dE\left( t \right)}{{d\left( t \right)}} = \alpha \beta_{2} S\left( t \right) + \gamma \varepsilon_{2} S\left( t \right) + b_{4} hR\left( t \right) - z_{1} E\left( t \right) - z_{2} E\left( t \right) - \left( {1 - z_{1} - z_{2} } \right)E\left( t \right) \\ \frac{{dI_{1} \left( t \right)}}{d\left( t \right)} = \alpha \beta_{1} S\left( t \right)I_{1} \left( t \right) + z_{1} E\left( t \right) + m_{2} xyI_{1} \left( t \right)I_{2} \left( t \right) + m_{3} xyR\left( t \right) - m_{1} \left( {1 - x} \right)\left( {1 - y} \right)I_{1} \left( t \right)I_{2} \left( t \right) - \left( {1 - b_{1} } \right)\left( {1 - q} \right)I_{1} \left( t \right) \\ \frac{{dI_{2} \left( t \right)}}{d\left( t \right)} = \gamma \varepsilon_{1} S\left( t \right)I_{2} \left( t \right) + z_{2} E\left( t \right) + m_{1} \left( {1 - x} \right)\left( {1 - y} \right)I_{1} \left( t \right)I_{2} \left( t \right) + m_{4} \left( {1 - x} \right)\left( {1 - y} \right)R\left( t \right) - m_{2} xyI_{1} \left( t \right)I_{2} \left( t \right) - b_{2} qI_{2} \left( t \right) \\ \frac{dR\left( t \right)}{{d\left( t \right)}} = \alpha \left( {1 - \beta_{1} - \beta_{2} } \right)S\left( t \right) + \gamma \left( {1 - \varepsilon_{1} - \varepsilon_{2} } \right)S\left( t \right) + \left( {1 - z_{1} - z_{2} } \right)E\left( t \right) + \left( {1 - b_{1} } \right)\left( {1 - q} \right)I_{1} \left( t \right) + b_{2} qI_{2} \left( t \right) - b_{4} hR\left( t \right) \\ \;\;\; - m_{3} xyR\left( t \right) - m_{4} \left( {1 - x} \right)\left( {1 - y} \right)R\left( t \right) \\ \end{gathered} \right. $$

The change rate of the density of the residents in the five states is represented by Eq. (1).

### Evolutionary game model

The transformation process among residents of each state in “Construction of the dissemination model” section is not only influenced by their own internal factors but also by the behavioral choices of the government and social capitals. Therefore, the tripartite game model of the government, social capitals and residents in GRs of TACs under the PPP model is constructed to describe the transformation process of residents' IGR under the influence of the behavioral choices of the government and social capitals. Residents are the users of GR PPP projects of TACs. The successful implementation of the project depends on the participation of the residents. Therefore, residents are included in the organizational framework of the PPP model. Under this model, the government, social capitals and residents jointly carry out GR, i.e., the three parties jointly invest, risk share and benefit distribute. Their respective investment costs and benefits are defined by signing a GR agreement. In particular, the government pays for the cost of GRs in the form of subsidies and obtains environmental benefits. Although both the government and social capitals want the project to run smoothly, they do not have the same goals. The government aims to maximize public benefits; social capitals aim to maximize their own benefits. Therefore, when the cost of government regulation is higher than the benefit, the government tends to choose not to regulate; when the benefit obtained by social capitals which do not effort to retrofit is higher than the effort to retrofit, they will have more opportunistic tendencies. It is important to note that residents are also core stakeholders. Whether or not the government regulates and whether or not social capitals effort are the main concern by residents, because the behavior of both the government and social capitals are closely related to the benefits of residents. Residents will consider the retrofit costs of participation and the loss of profit caused by social capitals' no effort. Therefore, the government must guide residents to participate by penalizing social capitals for their no effort behavior and compensating residents for their loss of benefits. Only when the government regulates, social capitals effort to retrofit, and residents participate, the performance of the project will improve and the tripartite will gain more potential benefits. Thus, a game relationship is formed between the three parties from mutual conflict to cooperation and win–win results.

#### Basic assumptions

##### Assumption 1

The government, social capitals and residents are all rational individuals. The strategy set of the government is {regulation, no regulation}, the strategy set of social capitals is {effort to retrofit, no effort to retrofit}, and the strategy set of residents is {participate and disseminate positive information, not participate and disseminate negative information, not pay attention and not disseminate}. The probability that the government chooses regulation is $x$ and on the contrary, it is $1 - x$. The probability that social capitals choose effort to retrofit is $y$ and on the contrary, it is $1 - y$. The probability that residents choose to participate and disseminate positive information, not to participate and disseminate negative information as well as not to pay attention nor to disseminate are $z_{1}$, $z_{2}$, $1 - z_{1} - z_{2}$, respectively.

##### Assumption 2

The government brings GRs of TACs into carbon trading mechanism. The carbon price is $r$, the free emission allowance allocated to the traditional apartment complex is $e$, and the initial emission volume of the traditional apartment complex is $e_{0} \left( {e_{{0}} > e} \right)$.

##### Assumption 3

The emission volume is taken as an important signal of whether social capitals make efforts to retrofit. Effort to retrofit mainly reflects that social capitals, by virtue of their talents and professional advantages, invest the retrofit cost $C_{2}$ as agreed to complete the target of retrofit, and the emission volume of the traditional apartment complex after retrofit is $e_{1} \left( {e_{1} < e} \right)$. No effort to retrofit mainly reflects that social capitals provide substandard services in order to obtain more benefits and reduce the input, and the retrofit cost is $C_{3} \left( {C_{2} > C_{3} } \right)$, resulting in the emission volume of the traditional apartment complex after retrofit is $e_{2} \left( {e_{2} > e} \right)$.

##### Assumption 4

GR projects can only be completed with the participation of residents on the consumer side. Therefore, when residents participate and social capitals effort to retrofit, social capitals and residents can distribute not only the base income of operating cost savings (social capitals and residents obtain $U_{1}$ and $U_{3}$, respectively), but also the income of excess carbon allowances sold $r\left( {e - e_{1} } \right)$. At this time, the proportion of income distribution from excess carbon allowances received by social capitals is $Q\left( {0 \le Q \le 1} \right)$, and the proportion received by residents is $1 - Q$; if social capitals no effort to retrofit, there is still a part of the income from operating cost savings obtained by the social capital and the residents (social capitals and residents obtain $U_{2}$ and $U_{4}$, respectively), but they need to share the cost of purchasing carbon allowances $r\left( {e_{2} - e} \right)$, and the proportion of cost sharing for social capitals and residents is $Q\left( {0 \le Q \le 1} \right)$, $1 - Q$, respectively. When residents choose not to participate in GRs, the behavior of social capitals' effort to retrofit will make them pay the cost of preliminary research $C_{{4}}$, but ultimately cannot realize the GR project, thus there is no benefit of GRs for the two parties and carbon trading is invalid.

##### Assumption 5

The government regulation refers to giving the subsidy $D_{1}$ to social capitals and giving the subsidy $D_{2}$ to residents when social capitals effort to retrofit and residents participate in GRs, charging the penalty $F_{1}$ to social capitals when social capitals do not effort to retrofit and paying the cost $C_{1}$ for regulating GRs. Whether the government regulates or not, when residents participate, if social capitals effort to retrofit, the government obtains environmental benefits $u\left( {e_{0} - e_{1} } \right)$ such as improved air quality and increased environmental capacity, where $u$ is the environmental benefit per unit of emission reduction; if social capitals do not effort to retrofit, the government obtains environmental benefits $u\left( {e_{0} - e_{2} } \right)$. When residents participate and social capitals effort to retrofit, if the government does not grant positive incentives such as subsidies, residents and social capitals will appeal to the government, and the group pressure and negotiation cost suffered by the government is set to $H_{1}$. When residents participate, social capitals choosing no effort to retrofit will suffer losses such as loss of customers and it is set to $F_{2}$, and the government choosing no regulation will suffer credibility losses, and it is set to $H_{2}$.

##### Assumption 6

When participating in GRs, residents need to pay the retrofit cost $C_{{5}}$. Social capitals' no effort to retrofit will damage the interests of the participating residents, and the residents obtain the damage compensation as $B$. The environmental awareness of residents is $b_{3} \left( {0 < b_{3} < 1} \right)$, and when residents participate in GRs, they can obtain the environmental awareness benefit $b_{3} U_{5}$. When residents disseminate information of GRs, they can improve their personal influence $V$, but when disseminating negative information of GRs, they will lose their reputation $W$.

According to the above assumptions, the game payoffs matrix of the government, social capitals and residents is obtained as shown in Table 2.

**Table 2:** Payoffs matrix for the government, social capitals and residents.

Residents	Government regulation $x$		Government no regulation $1 - x$	
	Social capitals effort to retrofit $y$	Social capitals no effort to retrofit $1 - y$	Social capitals effort to retrofit $y$	Social capitals no effort to retrofit $1 - y$
Participating and disseminating positive information $z_{1}$	$u\left( {e_{0} - e_{1} } \right) - D_{1} - D_{2} - C_{1}$	$$\begin{gathered} u\left( {e_{0} - e_{2} } \right) - D_{1} - \hfill \\ C_{1} - B + F_{1} \hfill \\ \end{gathered}$$	$u\left( {e_{0} - e_{1} } \right) - H_{1}$	$u\left( {e_{0} - e_{2} } \right) - H_{2}$
	$U_{1} + Qr\left( {e - e_{1} } \right) + D_{1} - C_{2}$	$$\begin{gathered} U_{2} - Qr\left( {e_{2} - e} \right) - \hfill \\ C_{3} - F_{1} - F_{2} \hfill \\ \end{gathered}$$	$U_{1} + Qr\left( {e - e_{1} } \right) - C_{2}$	$$\begin{gathered} U_{2} - Qr\left( {e_{2} - e} \right) - \hfill \\ C_{3} - F_{2} - B \hfill \\ \end{gathered}$$
	$$\begin{gathered} U_{3} + \left( {1 - Q} \right)r\left( {e - e_{1} } \right) + \hfill \\ D_{2} - C_{5} + V + W + b_{3} U_{5} \hfill \\ \end{gathered}$$	$$\begin{gathered} U_{4} - \left( {1 - Q} \right)r\left( {e_{2} - e} \right) + \hfill \\ D_{2} - C_{5} + V + W + \hfill \\ b_{3} U_{5} + B \hfill \\ \end{gathered}$$	$$\begin{gathered} U_{3} + \left( {1 - Q} \right)r\left( {e - e_{1} } \right) - \hfill \\ C_{5} + V + W + b_{3} U_{5} \hfill \\ \end{gathered}$$	$$\begin{gathered} U_{4} - \left( {1 - Q} \right)r\left( {e_{2} - e} \right) - \hfill \\ C_{5} + V + W + b_{3} U_{5} + B \hfill \\ \end{gathered}$$
No participating and disseminating negative information $z_{2}$	$0$	$0$	$0$	$0$
	$- C_{4}$	$0$	$- C_{4}$	$0$
	$V - W - b_{3} U_{5}$	$V - W - b_{3} U_{5}$	$V - W - b_{3} U_{5}$	$V - W - b_{3} U_{5}$
No paying attention and no disseminating $1 - z_{1} - z_{2}$	$0$	$0$	$0$	$0$
	$- C_{4}$	$0$	$- C_{4}$	$0$
	$- V - b_{3} U_{5}$	$- V - b_{3} U_{5}$	$- V - b_{3} U_{5}$	$- V - b_{3} U_{5}$

### Stability analysis of the tripartite evolutionary game

#### Construction of the replicated dynamic equation

The expected benefit when the government chooses the regulation strategy is $G_{11}$ and the expected benefit when the government chooses the no regulation strategy is $G_{12}$, the equations are shown below, respectively.

2 $$ G_{11} = yz_{1} \left[ {u\left( {e_{0} - e_{1} } \right) - D_{1} - D_{2} - C_{1} } \right] + \left( {1 - y} \right)z_{1} \left[ {u\left( {e_{0} - e_{2} } \right) - D_{2} - C_{1} - B + F_{1} } \right] $$

3 $$ G_{12} = yz_{1} \left[ {u\left( {e_{0} - e_{1} } \right) - H_{1} } \right] + \left( {1 - y} \right)z_{1} \left[ {u\left( {e_{0} - e_{2} } \right) - H_{2} } \right] $$

The replicated dynamic equation when the government chooses the regulation strategy is shown as follows.

4 $$ F\left( x \right) = x\left( {1 - x} \right)\left( {G_{11} - G_{12} } \right) = x\left( {1 - x} \right)\left[ {yz_{1} \left( { - D_{1} + H_{1} + B - F_{1} - H_{2} } \right) + z_{1} \left( { - D_{2} - C_{1} - B + F_{1} + H_{2} } \right)} \right] $$

In the same way, the replicated dynamic equation when social capitals choose the effort strategy is shown as follows.

5 $$ F\left( y \right) = y\left( {1 - y} \right)\left\{ {xz_{1} \left( {D_{1} + F_{1} - B} \right) + z_{1} \left[ {U_{1} - U_{2} + Qr\left( {e_{2} - e_{1} } \right) - C_{2} + C_{3} + C_{4} + F_{2} + B} \right] - C_{4} } \right\} $$

The replicated dynamic equation when residents choose to participate and disseminate positive information is shown as follows.

6 $$ F\left( {z_{1} } \right) = z_{1} \left\{ \begin{gathered} \left( {1 - z_{1} } \right)xD_{2} + \left( {1 - z_{1} } \right)y\left[ {U_{3} - U_{4} + \left( {1 - Q} \right)r\left( {e_{2} - e_{1} } \right) - B} \right] \hfill \\ + \left( {1 - z_{1} } \right)\left[ {U_{4} - \left( {1 - Q} \right)r\left( {e_{2} - e} \right) - C_{5} + 2V + W + 2b_{3} U_{5} + B} \right] - z_{2} \left( {2V - W} \right) \hfill \\ \end{gathered} \right\} $$

The replicated dynamic equation when residents choose not to participate and disseminate negative information is shown as follows.

7 $$ F\left( {z_{2} } \right) = z_{2} \left\{ \begin{gathered} \left( {1 - z_{2} } \right)\left( {2V - W} \right) - z_{1} xD_{2} - z_{1} y\left[ {U_{3} - U_{4} + \left( {1 - Q} \right)r\left( {e_{2} - e_{1} } \right) - B} \right] \hfill \\ - z_{1} \left[ {U_{4} - \left( {1 - Q} \right)r\left( {e_{2} - e} \right) - C_{5} + 2V + W + 2b_{3} U_{5} + B} \right] \hfill \\ \end{gathered} \right\} $$

#### Analysis of evolutionary stable points

Let $F\left( x \right) = F\left( y \right) = F\left( {z_{1} } \right) = F\left( {z_{2} } \right) = 0$, we can obtain 23 equilibrium points of the tripartite evolutionary game model, and there are 14 equilibrium points that satisfy ${0} \le x,y,z_{1} ,z_{2} \le 1$, which are $E_{1} \left( {0,0,0,0} \right)$, $E_{2} \left( {1,0,0,0} \right)$, $E_{3} \left( {0,1,0,0} \right)$, $E_{4} \left( {0,0,1,0} \right)$, $E_{5} \left( {0,0,0,1} \right)$, $E_{6} \left( {1,1,0,0} \right)$, $E_{7} \left( {1,0,1,0} \right)$, $E_{8} \left( {0,1,1,0} \right)$, $E_{9} \left( {1,0,0,1} \right)$, $E_{10} \left( {0,1,0,1} \right)$, $E_{11} \left( {1,1,1,0} \right)$, $E_{12} \left( {1,1,0,1} \right)$, $E_{13} \left( {1,\frac{{B - C_{5} + D_{2} + U_{4} + 2V + W + 2b_{3} U_{5} - \left( {1 - Q} \right)r\left( {e_{2} - e} \right)}}{{B - U_{3} + U_{4} - \left( {1 - Q} \right)r\left( {e_{2} - e_{1} } \right)}},\frac{{C_{4} }}{{C_{3} - C_{2} + C_{4} + D_{1} + F_{1} + F_{2} + U_{1} - U_{2} + Qr\left( {e_{2} - e_{1} } \right)}},0} \right)$, and $E_{14} \left( {1,\frac{{B - C_{5} + D_{2} + U_{4} + 2W + 2b_{3} U_{5} - \left( {1 - Q} \right)r\left( {e_{2} - e} \right)}}{{B - U_{3} + U_{4} - \left( {1 - Q} \right)r\left( {e_{2} - e_{1} } \right)}},\frac{{C_{4} }}{{C_{3} - C_{2} + C_{4} + D_{1} + F_{1} + F_{2} + U_{1} - U_{2} + Qr\left( {e_{2} - e_{1} } \right)}},\frac{{C_{3} - C_{2} + D_{1} + F_{1} + F_{2} + U_{1} - U_{2} + Qr\left( {e_{2} - e_{1} } \right)}}{{C_{3} - C_{2} { + }C_{4} + D_{1} + F_{1} + F_{2} + U_{1} - U_{2} + Qr\left( {e_{2} - e_{1} } \right)}}} \right)$.

These local equilibrium points are not certainly the stable points of the system, whose evolutionary stability can be judged by the Jacobi matrix of the system as shown in Eq. (8) ^78^. The analysis is carried out more intuitively by numerical simulation below due to the large number of parameters in the model.

8 $$ J = \left[ {\begin{array}{*{20}c} {\frac{\partial F\left( x \right)}{{\partial x}}} & {\frac{\partial F\left( x \right)}{{\partial y}}} & {\frac{\partial F\left( x \right)}{{\partial z_{1} }}} & {\frac{\partial F\left( x \right)}{{\partial z_{2} }}} \\ {\frac{\partial F\left( y \right)}{{\partial x}}} & {\frac{\partial F\left( y \right)}{{\partial y}}} & {\frac{\partial F\left( y \right)}{{\partial z_{1} }}} & {\frac{\partial F\left( y \right)}{{\partial z_{2} }}} \\ {\frac{{\partial F\left( {z_{1} } \right)}}{\partial x}} & {\frac{{\partial F\left( {z_{1} } \right)}}{\partial y}} & {\frac{{\partial F\left( {z_{1} } \right)}}{{\partial z_{1} }}} & {\frac{{\partial F\left( {z_{1} } \right)}}{{\partial z_{2} }}} \\ {\frac{{\partial F\left( {z_{2} } \right)}}{\partial x}} & {\frac{{\partial F\left( {z_{2} } \right)}}{\partial y}} & {\frac{{\partial F\left( {z_{2} } \right)}}{{\partial z_{1} }}} & {\frac{{\partial F\left( {z_{2} } \right)}}{{\partial z_{2} }}} \\ \end{array} } \right] $$

where $$\frac{\partial F\left( x \right)}{{\partial x}} = \left( {1 - 2x} \right)\left[ {yz_{1} \left( { - D_{1} + H_{1} + B - F_{1} - H_{2} } \right) + z_{1} \left( { - D_{2} - C_{1} - B + F_{1} + H_{2} } \right)} \right]$$, $$\frac{\partial F\left( x \right)}{{\partial y}} = x\left( {1 - x} \right)z_{1} \left( { - D_{1} + H_{1} + B - F_{1} - H_{2} } \right)$$, $$\frac{\partial F\left( x \right)}{{\partial z_{1} }} = x\left( {1 - x} \right)\left[ {y\left( { - D_{1} + H_{1} + B - F_{1} - H_{2} } \right) + \left( { - D_{2} - C_{1} - B + F_{1} + H_{2} } \right)} \right]$$, $$\frac{\partial F\left( x \right)}{{\partial z_{2} }} = 0$$, $$\frac{\partial F\left( y \right)}{{\partial x}} = y\left( {1 - y} \right)z_{1} \left( {D_{1} + F_{1} - B} \right)$$, $$\frac{\partial F\left( y \right)}{{\partial y}} = \left( {1 - 2y} \right)\left\{ {xz_{1} \left( {D_{1} + F_{1} - B} \right) + z_{1} \left[ {U_{1} - U_{2} + Qr\left( {e_{2} - e_{1} } \right) - C_{2} + C_{3} + C_{4} + F_{2} + B + C_{4} } \right] - C_{4} } \right\}$$, $$\frac{\partial F\left( y \right)}{{\partial z_{1} }} = y\left( {1 - y} \right)\left\{ {x\left( {D_{1} + F_{1} - B} \right) + \left[ {U_{1} - U_{2} + Qr\left( {e_{2} - e_{1} } \right) - C_{2} + C_{3} + C_{4} + F_{2} + B + C_{4} } \right]} \right\}$$, $$\frac{\partial F\left( y \right)}{{\partial z_{2} }} = 0$$, $$\frac{{\partial F\left( {z_{1} } \right)}}{\partial x} = z_{1} \left( {1 - z_{1} } \right)D_{2}$$, $$\frac{{\partial F\left( {z_{1} } \right)}}{\partial y} = z_{1} \left( {1 - z_{1} } \right)\left[ {U_{3} - U_{4} + \left( {1 - Q} \right)r\left( {e_{2} - e_{1} } \right) - B} \right]$$, $$\frac{{\partial F\left( {z_{1} } \right)}}{{\partial z_{1} }} = \left( {1 - 2z_{1} } \right)\left\{ {xD_{2} + y\left[ {U_{3} - U_{4} + \left( {1 - Q} \right)r\left( {e_{2} - e_{1} } \right) - B} \right] + U_{4} - \left( {1 - Q} \right)r\left( {e_{2} - e} \right) - C_{5} + 2V + W + 2b_{3} U_{5} + B} \right\} - z_{2} \left( {2V - W} \right)$$, $$\frac{{\partial F\left( {z_{1} } \right)}}{{\partial z_{2} }} = - z_{1} \left( {2V - W} \right)$$, $$\frac{{\partial F\left( {z_{2} } \right)}}{\partial x} = - z_{1} z_{2} D_{2}$$, $$\frac{{\partial F\left( {z_{2} } \right)}}{\partial y} = - z_{1} z_{2} \left[ {U_{3} - U_{4} + \left( {1 - Q} \right)r\left( {e_{2} - e_{1} } \right) - B} \right]$$, $$\frac{{\partial F\left( {z_{2} } \right)}}{{\partial z_{1} }} = - z_{2} \left\{ {xD_{2} + y\left[ {U_{3} - U_{4} + \left( {1 - Q} \right)r\left( {e_{2} - e_{1} } \right) - B} \right] + U_{4} - \left( {1 - Q} \right)r\left( {e_{2} - e} \right) - C_{5} + 2V + W + 2b_{3} U_{5} + B} \right\}$$, $$\frac{{\partial F\left( {z_{2} } \right)}}{{\partial z_{2} }} = \left( {1 - 2z_{2} } \right)\left( {2V - W} \right) - z_{1} \left\{ \begin{gathered} xD_{2} + y\left[ {U_{3} - U_{4} + \left( {1 - Q} \right)r\left( {e_{2} - e_{1} } \right) - B} \right] + U_{4} \hfill \\ - \left( {1 - Q} \right)r\left( {e_{2} - e} \right) - C_{5} + 2V + W + 2b_{3} U_{5} + B \hfill \\ \end{gathered} \right\}$$.

GRs of TACs require a joint vote by residents. Here we assume that the threshold value is 90%, namely, GRs can be carried out when the number of residents who agree to retrofit exceeds 90% of the number of residents who voted. Therefore, the densities of residents in the five states when tending to the steady state in the improved SEIR dissemination model constructed in this paper are used as indicators to measure whether the TAC can eventually be retrofitted. After receiving the information of GRs, the residents vote, the participating residents are considered to vote agree, the neutral residents are considered to vote neutral, and the resistant residents are considered to vote against. The immune residents do not participate in voting because they do not pay attention to and block the information of GR. Therefore, the immune residents are considered to vote invalid. Finally, the proportion of residents who agree to retrofits = the proportion of participating residents / the proportion of voting residents = $I_{1} \left( t \right)/\left( {1 - R\left( t \right)} \right)$. The following is to analyze the impact of parameters changes on the residents' IGR of TACs.

## Results and discussions

Referring to the related studies^33,77^, the proportion of different state residents at the initial time is $S\left( t \right) = 0.9$, $E\left( t \right) = 0$, $I_{1} \left( t \right) = 0.05$, $I_{2} \left( t \right) = 0.05$, and $R\left( t \right) = 0$, respectively. The initial values of $x$ and $y$ are 1/2, the initial values of $z_{1}$ and $z_{2}$ are 1/3, and the remaining parameters are taken as follows: $U_{1} = 25$, $U_{2} = 20$, $U_{3} = 15$, $U_{4} = 7$, $U_{5} = 5$, $b_{3} = 0.2$, $C_{1} = 6$, $C_{2} = 20$, $C_{3} = 5$, $C_{4} = 2$, $C_{5} = 12$, $D_{1} = 2$, $D_{2} = 2$, $B = 2$, $F_{1} = 5$, $F_{2} = 3$, $H_{1} = 15$, $H_{2} = 5$, $V = 0.5$, $W = 0.5$, $e = 8$, $e_{1} = 7$, $e_{2} = 9$, $Q = 0.5$, $r = 4$, $\alpha = 0.4$, $\beta_{1} = 0.5$, $\beta_{2} = 0.3$, $\gamma = 0.6$, $\varepsilon_{1} = 0.6$, $\varepsilon_{2} = 0.2$, $m_{1} = 0.5$, $m_{2} = 0.2$, $m_{3} = 0.5$, $m_{4} = 0.5$, $b_{1} = 0.6$, $b_{2} = 0.2$, $b_{4} = 0.5$, $q = 0.5$, $h = 0.8$.

**Figure 3:**
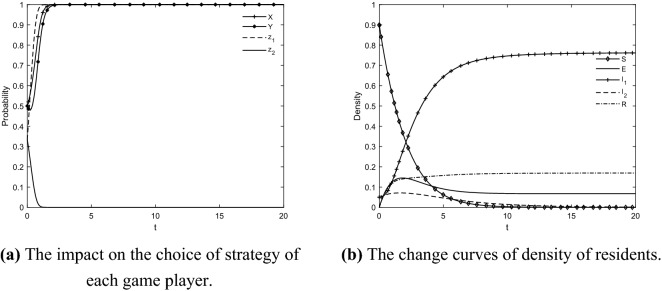
Initial diagram.

The initial state diagram can be obtained by inputting the settings of the above parameters into the simulation model, as shown in Fig. 3. It can be seen that the government, social capitals and neutral residents finally stabilize at $E_{11} \left( {1,1,1,0} \right)$. The transformation that occurs with different probabilities among the residents of each state is based on the behavioral choices of the government and social capitals. Initially, the information about GRs began to disseminate among resident groups, and residents gradually acquired the information, so the density of unknown residents rapidly decreased and reached zero, and the densities of neutral residents, participating residents, resistant residents, and immune residents began to increase. Positive and negative information exist simultaneously and interact with each other in the evolution process of residents' IGR. With the intervention of the government and social capitals, the dissemination of negative information among resident groups is weakened. Therefore, residents' intention as a whole moves toward the positive, which makes the density of participating residents dominate quickly, and its density curve is clearly on the upper right and stabilizes. On the contrary, the densities of resistant and neutral residents move from high to low, and their density curves reach a peak and then slowly decline and level off. There are always residents who lose interest in and attention to GRs in the dissemination process, so the density of immune residents has been slowly increasing and leveling off, and its density curve is maintained at a low but stable level. It can be obtained that the densities of unknown residents, neutral residents, participating residents, resistant residents, and immune residents under the synergistic effect of government regulation and social capitals' effort to retrofit are 0, 0.068, 0.762, 0.001, and 0.169 respectively, when the system reaches the equilibrium state. At this time, it can be obtained that the proportion of residents agreeing to GR of TACs = $I_{1} \left( t \right)/\left( {1 - R\left( t \right)} \right)$ = 92% > 90%, and finally the TAC is retrofitted.

### The impact of *C*_1_ on the choice of tripartite strategy and residents’ IGR

**Figure 4:**
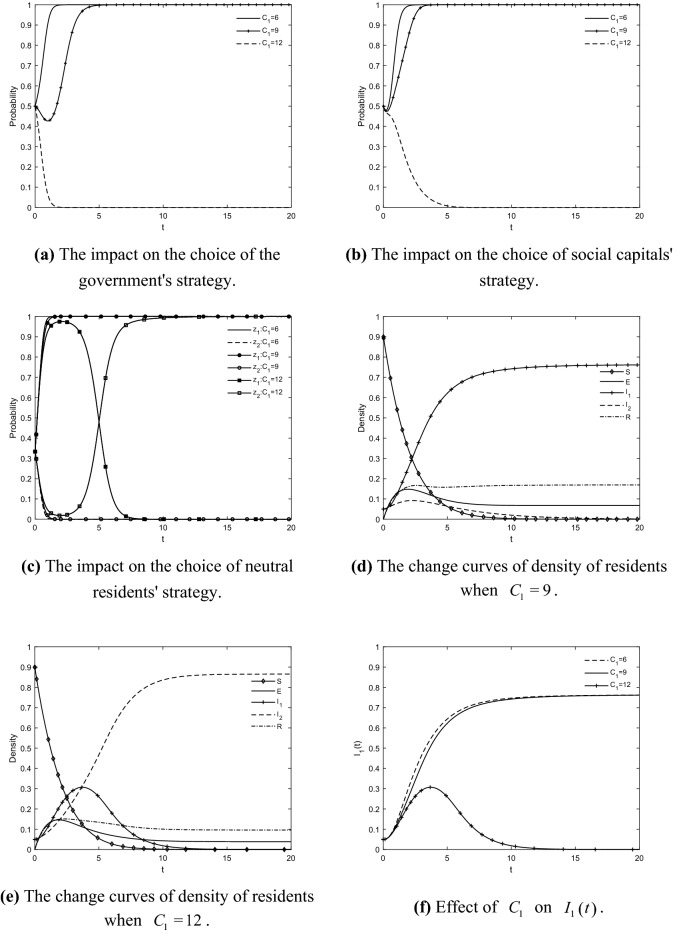
The impact of $C_{{1}}$ on the choice of tripartite strategy and residents' IGR.

Taking $C_{{1}} = 6$, $C_{{1}} = 9$ and $C_{{1}} = 12$ respectively, the evolutionary trajectories of the game players and the change curves of the density of residents in each state are obtained by simulation as shown in Fig. 4. The cost of government regulation significantly affects the evolutionary trend of the resident groups' IGR. When $C_{1} < 12$, the choice of strategies of the government, social capitals and neutral residents does not change, and the game system is all stable at $E_{11} \left( {1,1,1,0} \right)$. Thus, the density of residents of each state remains constant at this time when it tends to the steady state, but the speed at which the density curve of participating residents reaches its peak shortens with the speed at which the government and social capitals reach the steady state. When the cost of government regulation increases, there is a symmetric change in the size of the resistant and participating residents. The size of resistant resident increases significantly, the peak of the density curve of participating resident decreases and the density curve rapidly tends to zero. The main reason is that the benefits of government regulation are smaller than the benefits of no regulation, and social capitals tend to choose no effort to retrofit in the absence of effective government constraints. Therefore, the negative behavior of the government and social capitals leads to a high negative sentiment of residents, and the probability of participating residents and immune residents transforming into resistant residents increases, and the size of resistant residents climbs significantly and gradually exceeds that of participating residents. In the end, the number of residents who agree is zero. It is unable to realize the GR of TACs. Thus, it can be seen that the cost of government regulation affects the strategic choice of government and social capitals in the first place. Moreover, because the government chooses no regulation and social capitals choose no effort to retrofit, the acceptance of GRs by residents decreases and there is an extreme phenomenon of negative emotion eruption among the resident groups. It can be inferred that the higher the cost of government regulation, the less conducive to promoting GRs. Reducing the cost of government regulation not only improves the initiative of government regulation and restrains the behavior of social capitals, but also helps to increase the trust of residents in the government and social capitals and minimizes the diffusion of resistance among the group. Therefore, in order to form effective cooperation with the residents as soon as possible, the government should reduce the cost of regulation by improving the supervision and inspection mechanisms.

### The impact of *D*_2_ on the choice of tripartite strategy and residents' IGR

**Figure 5:**
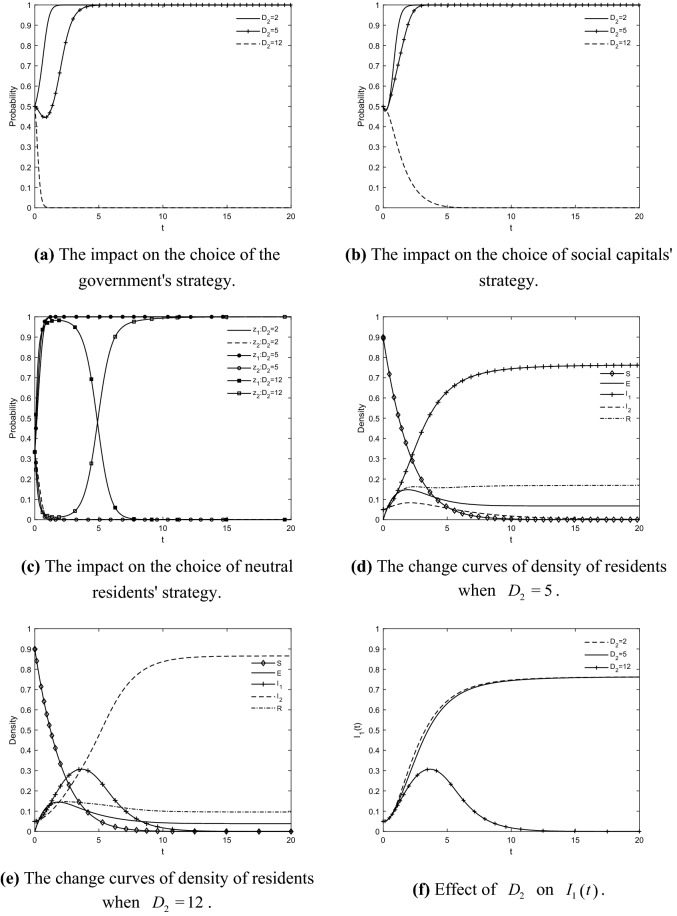
The impact of $D_{{2}}$ on the choice of tripartite strategy and residents' IGR.

The evolutionary trajectories of the game players and the change curves of the density of residents in each state are obtained by taking $D_{2} = {2}$, $D_{2} = {5}$ and $D_{2} = 1{2}$, respectively, as shown in Fig. 5. When the subsidy decreases, the density of residents in each state eventually converges to the same result. However, as government subsidy increases, the resident group's IGR significantly changes. When $D_{2} = 1{2}$, the density curve of participating residents shows a rapid increase and then accelerated decrease towards zero. On the contrary, the density of resistant resident increases rapidly and gradually prevails over time. This indicates that the government will choose to abandon regulatory behavior when its finances can hardly support the over-invested subsidy. In turn, residents without financial incentives gradually lose interest in participation and inhibit the diffusion of positive information and the rapid spread of negative emotions in the group. As a result, the proportion of participating resident decreases significantly, the proportion of resistant residents becomes larger and larger, and the situation is even "lopsided", so that the number of residents agreeing to the retrofit cannot be reached. It is clear that positive incentive mechanisms such as the subsidy reasonably implemented by the government for residents are essential to mobilize their participation and realize the GR of TACs. However, when the subsidy exceeds a certain range, it rather stalls residents' enthusiasm to participate and breaks the stability of the system, because the government will choose not to regulate based on financial pressure. This shows that moderate subsidy can reduce economic burden and motivate participation of residents, but excessive subsidy can also have the negative effect of failing to maintain the stability of GRs. In other words, an effective incentive mechanism is necessary to improve the motivation of residents to retrofit. Therefore, to ensure that residents' intention to participate is not discouraged, the government should guarantee the overall benefit while giving the subsidy to residents.

### The impact of *F*_1_ on the choice of tripartite strategy and residents' IGR

**Figure 6:**
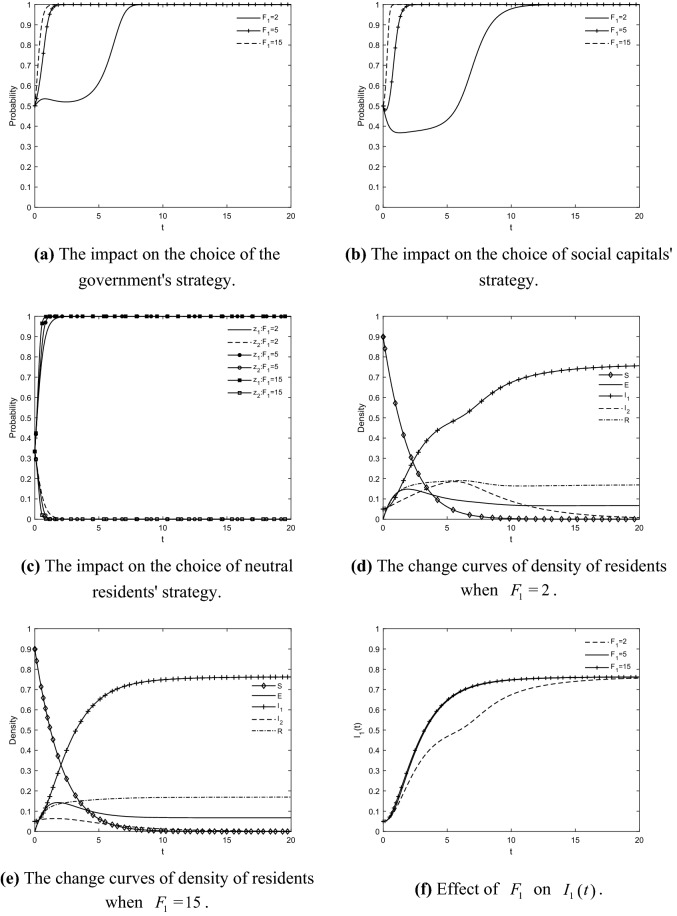
The impact of $F_{{1}}$ on the choice of tripartite strategy and residents' IGR.

Taking $F_{1} = {2}$, $F_{1} = {5}$ and $F_{1} = {15}$ respectively, the evolutionary trajectories of the game players and the change curves of the density of residents in each state are obtained as shown in Fig. 6. The stronger the government's punitive measures against social capitals that do not effort to retrofit, the faster social capitals converge on the effort to retrofit strategy, which contributes to the faster the density of participating residents increases and stabilizes. When $F_{1} = {2}$, the slow rate at which the probability of government regulation and the probability of social capitals' effort to retrofit converge to 1 significantly accelerates the diffusion of negative information among residents. This leads to a slow increase in the density of participating residents over time and even a sudden increase in the density of resistant residents in the early period. The reason is that the motivation of government regulation and social capitals' effort to retrofit affects the probability of transforming resistant residents and immune residents into participating residents. It can be seen that stronger government penalties can effectively shorten the time for the density curve of participating residents to reach its peak, and also reduce the peak of the density of resistant and immune residents by inhibiting the spread and deterioration of resistance in time, which enables a shorter time required to realize the GR of TACs. The stronger the penalty the more effective the government is in restraining the opportunistic behavior of social capitals, which in turn protects the rights of residents from being harmed. As a result, residents' sentiment is calmed and the peak of the density curve of resistant residents appears to fall back. Too low a penalty will increase the risk that residents will not choose to cooperate with social capitals because of inadequate government regulation. It can be inferred that the series of penalties adopted by the government can deter social capitals and thus weaken the risks involved in GRs, which is favorable to promote GR and improve the implementation effect of GRs. Therefore, it is important to increase the penalty for the speculative behavior of social capitals to prevent residents from being incited by malicious information.

### The impact of *r* on the choice of tripartite strategy and residents' IGR

**Figure 7:**
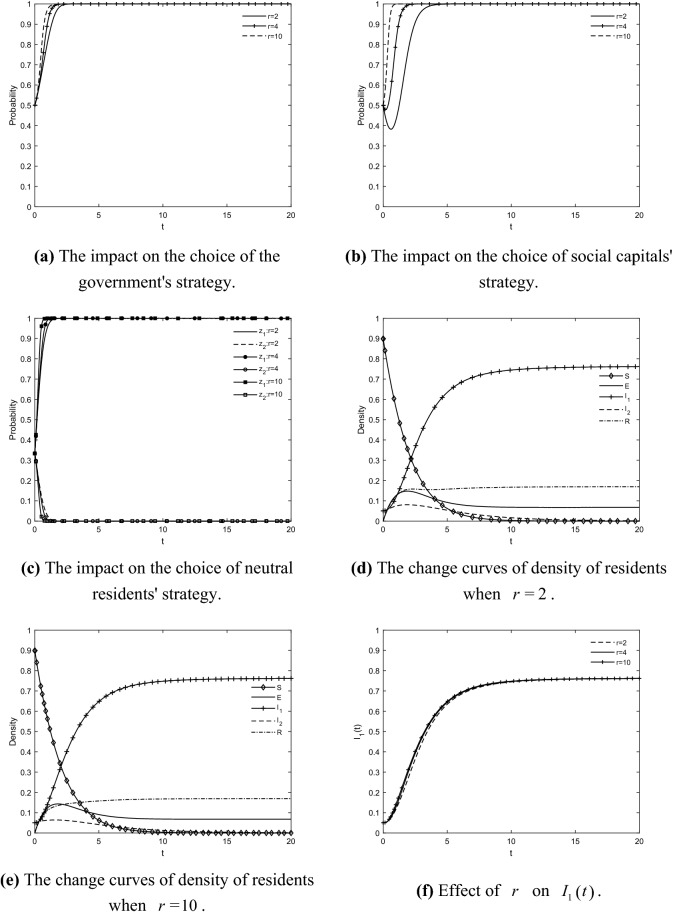
The impact of $r$ on the choice of tripartite strategy and residents' IGR.

Taking $r = {2}$, $r = {4}$ and $r = {10}$ respectively, the evolutionary trajectories of the game players and the change curves of the density of residents in each state are obtained as shown in Fig. 7. By increasing the carbon price, the game players all evolve in a positive direction and the overall change trend of residents in each state remains basically the same, but the density of participating residents grows faster and converges earlier. This is because the increase in the carbon price obviously increases the profits of social capitals' effort to retrofit and residents' participation, they are able to profit through carbon trading while gaining energy-saving benefits. This indicates that the greater the benefits of residents' participation in GR projects, the stronger the residents' intention to participate, which is also consistent with the research law of some scholars^11,12^. It can be seen that economic benefits influence residents' IGR. The higher the carbon price, the more positive the intention of the resident group and the more easily the residents' resistance can be eliminated, prompting the residents in TACs to reach a consensus on GR earlier. In reality, the externalities of GRs weaken the demand of residents for retrofits because they prefer to pursue economic benefits. Based on this, TACs can be included in the carbon trading market, and this market mechanism can be used to motivate profit-oriented residents who tend to take more responsibility for emission reduction. Therefore, with the help of the carbon trading mechanism, the government can increase the enthusiasm of residents for GRs and at the same time reduce carbon emissions. Thus, it can be thought that the carbon trading mechanism is an important policy tool to promote the GR of TACs and to achieve both economic development and environmental protection.

### The impact of *q* on the residents’ IGR

**Figure 8:**
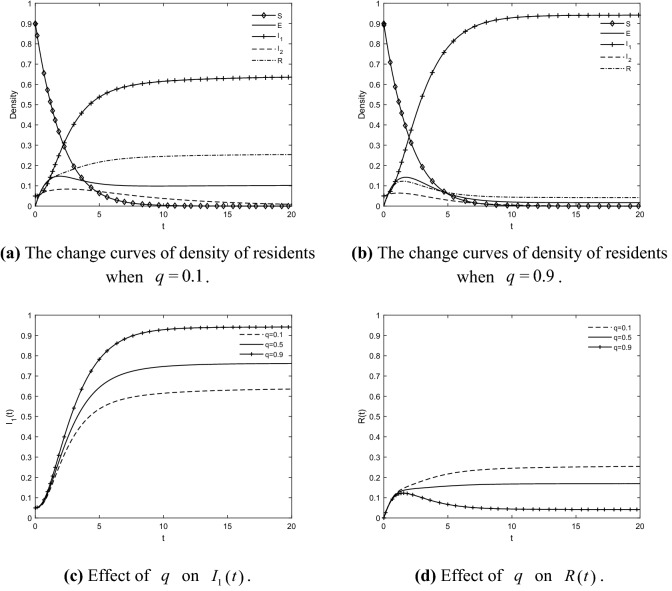
The impact of $q$ on the residents' IGR.

Taking $q = {0}{\text{.1}}$, $q = {0}{\text{.5}}$ and $q = {0}{\text{.9}}$ respectively, the change curves of the density of residents in each state are obtained as shown in Fig. 8. When $q = {0}{\text{.1}}$, the densities of participating and immune residents when they reach the steady state are 0.638 and 0.255, respectively, which can be derived from the proportion of residents who agree to the GR of TACs = $I_{1} \left( t \right)/\left( {1 - R\left( t \right)} \right)$ = 86% < 90%, which does not meet the minimum percentage requirement for the GR. As the level of government publicity and education improves, the density of participating resident increases substantially, the density curve of resistant residents tends to zero slightly earlier, the density of neutral residents also decreases to some extent, and the density of immune residents peaks less and less. When $q = {0}{\text{.9}}$, the densities of participating and immune residents when they reach a stable state are 0.941 and 0.041, respectively, and the proportion of residents who agree to the GR of TACs can be obtained = $I_{1} \left( t \right)/\left( {1 - R\left( t \right)} \right)$ = 98% > 90%. It can be seen that the residents' intention to participate is influenced by the level of government publicity and education. With the effective guidance and communication of the government at the early stage of disseminating GR, it is easier for residents to obtain positive information and eliminate residents' sensitivity, which is conducive to creating a good atmosphere for retrofit, and prompting the resident group's intention to develop a positive posture. This shows that the environmental value orientation and responsibility awareness of residents can be effectively guided and strengthened by improving the level of government publicity and education. With negative information being maliciously disseminated, residents have a more rational judgment of negative information; with positive information being actively disseminated, GR PPP projects receive high attention from residents and stimulate their interest in participation. Thus, it can be believed that the government's publicity and education is necessary to guarantee the good running of the market, as it can guide the overall intention of residents to be positive. Therefore, the government must pay attention to publicity and education to achieve a higher sense of responsibility among residents and to make GRs gain widespread support from them.

## Conclusions and recommendations

Whether the number of residents agreeing to GR of TACs can reach the proportional requirement is an important part of implementing GR PPP projects. This paper focuses on the resident group, and takes the interaction among individual residents and the interaction of multi-subject behavior as the perspective, and firstly constructs the dissemination model of residents' IGR, and couples the dissemination model with the evolutionary game model of the government, social capitals and residents under the GR PPP model. The influence of the dynamic evolution process of the strategies of the government and social capitals and the evolution trend of the resident group's IGR under the change of relevant factors is analyzed. The results are as follows.

1. The choice process of the government and social capitals' strategies has a direct impact on the evolutionary trend of residents' IGR of TACs. The behavior of government regulation and social capitals' effort to retrofit promote the number of residents who agree to the GR to meet the proportional limit and realize the GR of TACs. The behavior that the government does not regulate and the social capitals do not effort to retrofit makes the residents' IGR evolve to the opposite situation, that is, the phenomenon that the proportion of participating residents decreases greatly and the scale of resistant residents increases significantly, resulting in the proportion of residents failing to meet the requirements and finally failing to achieve retrofit. Therefore, the government and social capitals should play a linkage role to promote the behavior of the three parties towards a good posture.
2. The speed at which the government chooses the strategy of regulation and the social capitals choose the strategy of effort to retrofit affects the speed at which the residents of each state reach the steady state. The faster the government chooses the strategy of regulation and the social capitals choose the strategy of effort to retrofit, the faster the residents of each state reach the stable state and the shorter the time for the resident group to agree to GR. By increasing the penalties for social capitals which do no effort to retrofit, the government can not only restrain the behavior of social capitals but also effectively attract the participation of residents. Therefore, to achieve GRs of TACs, an adjustment of reward and punishment mechanisms can be implemented by the government.
3. The regulatory cost of the government and the subsidy given to residents by the government play a decisive role in the evolutionary trend of residents' IGR. The higher the cost of government regulation and the more the subsidy given to residents, the more the government chooses not to regulate because of the incongruity between input and output. This will lead to the dissemination of negative emotions among residents and eventually fail to meet the requirement of the proportion of residents who agree to the retrofit. Therefore, in order to promote tripartite cooperation, the government should reasonably regulate and subsidize.
4. The carbon price has an important impact on the evolution of the residents' IGR. The more the carbon price is increased, the more the residents gain from participating in the retrofit and the faster the transformation into participating residents. Therefore, a flexible regulation of the carbon price is beneficial to motivate residents to participate in the retrofit.
5. The evolutionary trend of the resident group's IGR is influenced not only by the behavioral choices of the government and social capitals, but also by the level of government publicity and education. When improving the government's publicity and education level, the density of participating resident increases and the density of immune resident decreases, so as to realize the change from non-retrofit to retrofit. Therefore, the government should go deeper into the resident groups, strengthen the communication with residents, actively popularize the knowledge of GRs to residents, and create a green atmosphere to achieve the GR of TACs.

Based on the above discussion, it is clear that in achieving GRs of TACs, the government plays an irreplaceable role as a policy maker, an implementer of public decisions and a supervisor of market players. First, it is important to pay attention to the participation of important players such as industry associations, the media and the public and the use of the Blockchain, the Internet of Things and other technologies to monitor the GR process in real time, so as to assist the government in regulating the market. This will not only save the government's regulatory costs, but also dispel the luckiness of social capitals. Second, for social capitals that actively retrofit, the government can establish subsidy incentive mechanisms or introduce the carbon trading mechanism. The retrofit area and emission reduction volume will be used as the evaluation criteria to provide direct or indirect support to social capitals and residents. However, it is necessary to avoid negative effects such as regulatory ineffectiveness due to the high subsidy. For social capitals that do not actively retrofit, the punishment by the government for this behavior should be increased appropriately. In addition to direct penalty, scoring and credit rating mechanisms can be established at each stage of the retrofit, and the social image of social capitals will be seriously affected in a negative behavior. Third, the government should simplify the approval process of GR projects to reduce the cost of participating parties. Fourth, fostering environmental awareness among residents should be given attention by the government. In order to correct the misconceptions of residents about GR, effectively alleviate their resistance and timely control their dissemination of negative information, the following approaches can be adopted: deepening the environmental experience of residents through outdoor teaching, guiding the concepts of environmentally friendly living through learning and education in schools and green lifestyles in families, and spreading the knowledge of GRs to residents through distributing environmental guidelines and posting advertisements. Fifth, in order to promote the development of the market on a large scale, the experience of GRs can be replicated and diffused from good pilot projects. Sixth, upgrading the technology of GRs for social capitals is the priority. For example, the key technology can be upgraded through independent innovation, or through cooperative innovation, that is, with the help of the talents and resources of universities to provide impetus for social capitals to develop innovative technology. This can not only reduce the cost of investment but also increase the benefits of the project to increase the confidence of the residents to gain profit.

The limitations of the paper are: (1) The synergistic or offsetting interactions between different factors are ignored in the dissemination model of residents' IGR of TACs, and further improvements can be made to the model in the future. (2) The GR market of TACs in China has different characteristics based on the differences in climate, culture and customs between the north and the south of China, and the model can be verified and improved by obtaining real data through field research in the future.

## Data Availability

All data generated or analyzed during this study are included in this published article.
